# Visual Identification and Serotyping of Toxigenic *Vibrio cholerae* Serogroups O1 and O139 With CARID

**DOI:** 10.3389/fcimb.2022.863435

**Published:** 2022-03-31

**Authors:** Pan Lu, Jialiang Chen, Zhenpeng Li, Zhe Li, Jingyun Zhang, Biao Kan, Bo Pang

**Affiliations:** State Key Laboratory of Infectious Disease Prevention and Control, National Institute for Communicable Disease Control and Prevention, Chinese Center for Disease Control and Prevention, Beijing, China

**Keywords:** CARID, CRISPR-Cas, RAA, detection, *Vibrio cholerae*, cholera

## Abstract

There is a growing demand for rapid, sensitive, field-deployable nucleic acid tests for cholera, which usually occurs in rural areas. In this study, we developed a Cas12a-assisted rapid isothermal detection (CARID) system for the detection of toxigenic *V. cholerae* serogroups O1 and O139 by combining recombinase-aided amplification and CRISPR-Cas (clustered regularly interspaced short palindromic repeats and CRISPR-associated proteins). The results can be determined by fluorescence signal and visualized by lateral flow dipstick. We identified 154 V*. cholerae* strains and 129 strains of other intestinal diarrheagenic bacteria with a 100% coincidence rate. The limit of detection of CARID was 20 copies/reaction of *V. cholerae* genomic DNA, which is comparable to that of polymerase chain reaction (PCR) and qPCR. Multiple-CARID was also established for efficiency and economic considerations with an acceptable decrease in sensitivity. Simulated sample tests showed that CARID is suitable for complex samples. In conclusion, CARID is a rapid, sensitive, economically efficient, and portable method for the detection of *V. cholerae*, which makes it suitable for field responses to cholera.

## Introduction

Cholera remains a threat to public health in many countries with poor sanitation or that are short of safe water (Deen et al., 2020). It has been estimated that there are roughly 1.3 to 4.0 million cases of cholera annually, with 21,000 to 143,000 deaths worldwide (Ali et al., 2015). *Vibrio cholerae*, the causative agent of cholera, can be transmitted through contaminated water and/or food (Sack et al., 2004). Cholera toxin (CT), an important pathogenic factor of *V. cholerae*, is encoded by *ctxA* and *ctxB*, and *V. cholerae* that carry *ctxA* and *ctxB* are referred to as toxigenic strains (Bharati and Ganguly, 2011). To date, only the toxigenic serogroups O1 and O139 are known to have caused epidemics and pandemics of cholera, even though there are more than 200 serogroups of *V. cholerae* (Harris et al., 2012; Chowdhury et al., 2017).

Early detection and confirmation of cholera cases are critical for rapid implementation of interventions. However, traditional culture methods for isolation and identification of *V. cholerae* can take three days or more and require extensive laboratory infrastructure and experienced staff (Ramamurthy et al., 2020). While molecular methods such as polymerase chain reaction (PCR) and quantitative real-time PCR (qPCR) have accelerated the diagnosis process, they rely heavily on expensive instruments and require elaborate experimental conditions, which are often difficult to obtain in rural areas. Therefore, rapid and point-of-care testing methods are essential for timely cholera detection and control.

CRISPR-Cas (clustered regularly interspaced short palindromic repeats and CRISPR-associated proteins) systems are adaptive immune systems consisting of Cas effector proteins and CRISPR RNAs (crRNAs) that are widely distributed in archaea and bacteria (Li and Du, 2011; Terns and Terns, 2011). CRISPR-Cas relies on crRNAs for sequence-specific detection and silencing of foreign nucleic acids, thereby protecting organisms from viruses and phages (Barrangou et al., 2007; Al-Attar et al., 2011; Bhaya et al., 2011; Wiedenheft et al., 2012). CRISPR-Cas12a systems can cleave single-stranded DNA (ssDNA) indiscriminately when bound to target sequences under the guidance of crRNA *in vitro* (Chen et al., 2018); therefore, this principle has been used in the specific detection of pathogens (Chen et al., 2018; Gootenberg et al., 2018; Li et al., 2018). For double-stranded target DNA activators, the prerequisite for Cas12a to effectively cleave non-target DNA is identification of a short T-rich (5 ‘-TTTN-3’) protospacer-adjacent motif (PAM) in the target strand (Zetsche et al., 2015; Chen et al., 2018), which provides theoretical guidance for the design of crRNA.

Because the detection ability of CRISPR-Cas systems relies on the template provided, it is usually combined with several amplification methods, such as recombinase polymerase amplification (RPA) (Chen et al., 2018), PCR (Li et al., 2018) and recombinase-aided amplification (RAA) (Xiao et al., 2021). RAA is a sensitive, rapid, low-cost isothermal amplification technique that can be completed within 15–30 min at 37 to 42°C without using large, expensive instruments (Shen et al., 2019; Wang et al., 2020).

**Figure 1:**
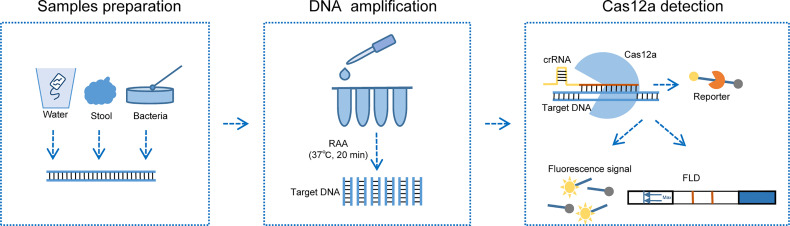
Schematic diagram of CARID detecting *V. cholerae*. Nucleic acid can be extracted from *V. cholerae* contaminated river water samples, stool, and clones cultured on LB agar plates. RAA amplifies the template at 37°C for 20 min, after which it is added to the Cas12a reaction mixture and incubated at 37°C for 30 min. The fluorescent signal and lateral flow dipstick can be used to determine the results.

Based on the detection principles of CRISPR-Cas12a and RAA, we developed CARID (Cas12a-assisted rapid isothermal detection) for the rapid visual detection of toxigenic *V. cholerae* serogroups O1 and O139 (**Figure 1**). Using simulated samples, we demonstrated that CARID could be completed within an hour, and that the results could be visualized through fluorescent signals or lateral flow dipstick (LFD). The method established in this study can detect the *ctxA* gene and identify the O1 and O139 serogroups with a specificity of 100%. The minimum detectable genomic concentration is 20 copies/reaction, and the sensitivity is comparable to that of PCR and qPCR.

## Materials and Methods

### Bacterial Strains

A total of 154 strains of *V. cholerae* were included in this study, namely, 112 strains of *V. cholerae* carrying cholera toxin (22 strains of serogroup O1 and 90 strains of serogroup O139) and 42 strains of *V. cholerae* without CT (22 strains of serogroup O1 and 20 strains of serogroup O139). These strains were isolated from cholera patients and environmental samples. Oral consent was obtained from each eligible patient (or the legal guardian of the patient, if the patient is <18 years of age). Other intestinal diarrheagenic bacteria, namely, 20 strains of *Vibrio parahaemolyticus*, 14 strains of *Vibrio alginolyticus*, 13 strains of *Vibrio mimicus*, 14 strains of *Vibrio furnis*, 10 strains of *Vibrio fluvialis*, 14 strains of *Escherichia coli*, 15 strains of *Salmonella enteritidis*, 14 strains of *Shigella*, and 15 strains of *Yersinia enterocolitica* were also evaluated in this study.

### Purification of LbCas12a Protein

To prepare recombinant LbCas12a proteins (Zetsche et al., 2015), protein sequences were codon-optimized for *E. coli* expression. Synthetic oligonucleotide fragments were then cloned into bacterial protein expression vector pET-28a and transfected into *E. coli* BL21. *E. coli* cells were first grown with 50 µg/ml Kanamycin at 37°C until OD_600_ reached 0.5–0.6, followed by incubation with 0.5 mM IPTG at 21°C for 16 h to induce LbCas12a expression. The cultures were then lysed by sonication, filtered through 0.22-µm filters, and applied to a nickel column (Ni-NTA agarose, Qiagen, Germany) for protein purification. The eluted protein was stored in a storage solution (500 mM NaCl, 20 mM Sodium Acetate, 0.1 mM EDTA, 0.1 mM DTT, 50% Glycerol). Purified LbCas12a proteins were examined by SDS-PAGE and protein concentrations were determined using a Pierce™ BCA Protein Assay Kit (Thermo Scientific, Shanghai, China).

### Specificity Determination of CARID

DNA was extracted from bacterial colonies cultured overnight using the boiling method, after which the extracted nucleic acid was dissolved in 50 µl ddH_2_O. Target DNA was amplified using an RAA isothermal amplification kit (Qitian, Jiangsu, China) followed by Cas12a detection. Briefly, 50 µl RAA reactions contained 2 µl template, 0.4 µM forward and reverse primer, 1× reaction buffer, nuclease-free water, lyophilized powder reaction unit, and 2.5 µl of 280 mM magnesium acetate. The RAA mix was incubated at 37°C for 20 min. For the fluorescence assay, the amplification product was added to CRISPR reaction mix consisting of 1× CRISPR reaction buffer (10 mM Tris–HCl, 10 mM MgCl_2_, pH = 8.0), 100 nM crRNA, 100 nM Cas12a, and 200 nM ssDNA reporter (5’-6FAM-TTATT-BHQI-3’). This final reaction was incubated at 37°C and monitored for fluorescence for 30 min using a Gentier 96E qPCR machine (TIANLONG, Xian, China). Samples that produced fluorescence at levels 2.0-fold or greater above the NC were considered positive. For the LFD detection reactions, the ssDNA reporter was replaced with 5’-6FAM-TTATT-Biotin-3’. The final reaction system was incubated at 37°C for 30 min, after which the reaction products were diluted with ddH_2_O and then inserted into the LFD (Tiosbio, Beijing, China) to read the results. A band on the T test line can be judged as positive.

### Sensitivity of CARID

To evaluate the sensitivity of CARID, *V. cholerae* genomic DNA was extracted using a Wizard Genomic DNA Purification Kit (Promega, Madison, WI, USA) and diluted with ddH_2_O to concentrations of 10^7^ to 1 copies/µl (DNA copies number were determined using following formula: (6.02 ×  10^23^) × (ng/µl  × 10^−9^)/(DNA length × 660) = copies/µl). Diluted samples were then used as templates for CARID detection, PCR, and qPCR. PCR was conducted using TaKaRa Premix Taq™ (TaKaRa, Dalian, China) and a 50-µL reaction system that contained 1×Premix Taq, 0.4 µM forward primer, 0.4 µM reverse primer, and 2 µl of template. The reaction conditions were 95°C for 5 min, followed by 40 cycles of 95°C for 10 s, 50°C for 30 s, and 72°C for 30 s, and then 72°C for 10 min. A qPCR assay was performed in a 50-µl volume using Premix Ex TaqTM (TaKaRa, Dalian, China). The reaction system contained 1× Premix Ex Taq (Probe qPCR), 0.4 µM forward primer, 0.4 µM reverse primer, 0.4 µM probe, and 2 µl of template. The reaction conditions for qPCR were 95°C for 30 s, followed by 40 cycles of 95°C for 10 s and 60°C for 30 s. The amplification process was conducted using a Gentier 96E qPCR machine (Tianlong, Xian, China).

### Verification of CARID With Simulated Samples

#### Verification of CARID With Simulated Environmental Water Samples

One toxigenic serogroup O1 and one toxigenic serogroup O139 of *V. cholerae* was cultured on nutrient agar overnight. Individual colonies were then selected and incubated in Luria–Bertani (LB) broth (Oxoid, Basingstoke, UK) at 37°C with shaking (200 rpm) to a concentration of OD_600_ = 1.0 (approximately 1 × 10^9^ colony forming units (CFU)/ml), after which the bacteria were gradient-diluted into a bacterial suspension in PBS with a concentration of 10^9^–10 CFU/ml. Simulated water samples with bacterial concentrations of 10^7^–10^−1^ CFU/ml were prepared by adding 50 µl of bacterial suspension to 5 ml of pre-mixed and sterilized river water (4.5 ml of river water and 0.5 ml of 10× 1% sodium chloride alkaline peptone water). The simulated water samples were then shaken at 200 rpm and 37°C for 4 h, and 1 ml of bacterial suspension was taken every hour for nucleic acid extraction and CARID detection as described above. Fluorescent signal detection and LFD were then used to visualize the results.

#### Verification of CARID With Simulated Stool Samples

The bacterial deposits of 10^9^–10 CFU were mixed with 250-mg stool samples of healthy adults, after which nucleic acid was extracted using a QIAamp^®^ Power Fecal^®^ Pro DNA Kit (Qiagen, Germany) according to the manufacturer instructions. The extracted nucleic acid was then dissolved in 50 µl ddH_2_O, after which 2-µl aliquots were used as templates for detection of the *ctxA* and O-PS specific genes. Fluorescent signal detection and LFD were used to visualize the results.

### Identification of *V. cholerae* With Multiple CARID

The multiple RAA system included 1× reaction buffer, 0.4 µM forward and reverse primers for *V. cholerae ctxA*, serogroups O1 and O139 O-PS specific genes, and the RAA reagent. After preparing the system, 2 µl of DNA template and 2.5 µl of 280 mM magnesium acetate solution were added and samples were then incubated at 37°C for 30 min. CrRNA was then used to detect *ctxA*, O1, and O139 O-PS specific genes in the product. Fluorescent signal detection and LFD were used to visualize the results.

### Statistical Analysis

The GraphPad Prism 9 software was used for statistical analyses and preparation of figures. Analyses were based on three replicate values. Unpaired t-tests were used to identify differences between two groups.

## Results

### Design of Primers and crRNA

Primers and crRNA were designed according to the conserved regions of *ctxA*, rfb-O1, and rfb-O139 genes of *V. cholerae*. Conserved fragments containing the PAM sequence (5’-TTTN-3’) were selected as the target to design primers and guide sequences. CrRNA contains a conserved stem-loop structure that is necessary for forming the Cas12a–crRNA complex, and the adaptive crRNA stem-loop sequence of Cas12a is different for each Cas12a type (Zetsche et al., 2015). After the guide sequence was designed, a stem-loop sequence homologous to LbCas12a was added to its 5’ end to ensure that the protein had strong cleavage ability. The primers and RNA sequences are not published herein because they are currently being patented.

### Validation of LbCas12a Protein Activity and crRNA Adaptation

**Figure 2:**
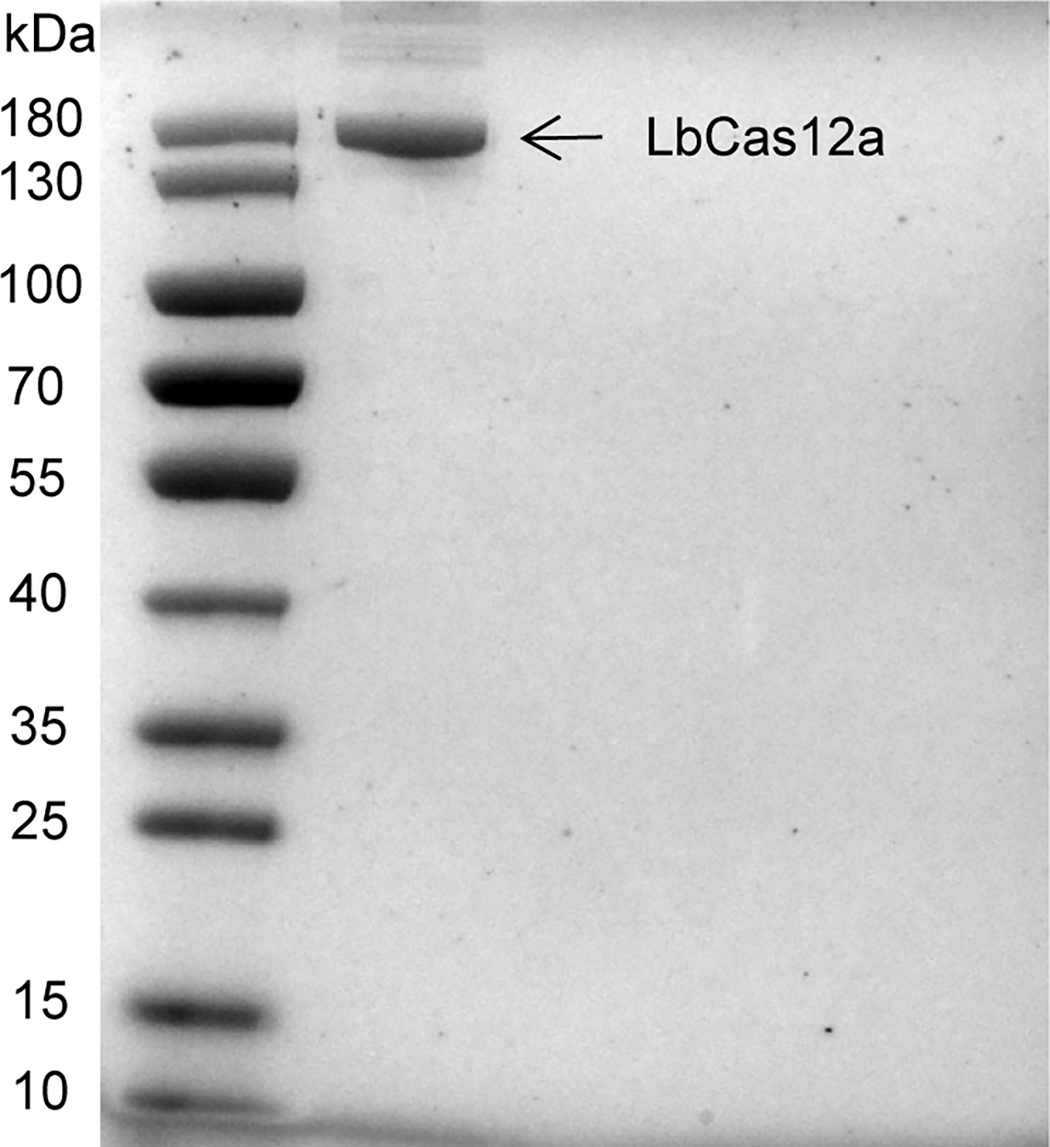
Purification of Cas12 proteins. SDS-PAGE gel of LbCas12 proteins was used in this study.

**Figure 3:**
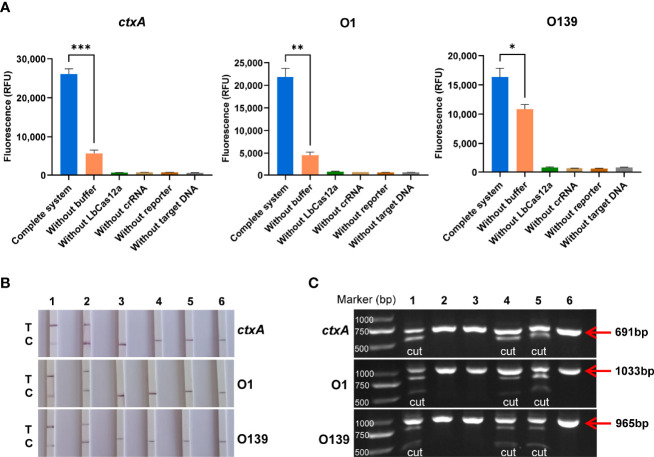
Validation of LbCas12a protein activity and crRNA adaptation. **(A)** Fluorescence value of the complete Cas12a system, and the absence of buffer, Cas12a, crRNA, reporter or target DNA (n = 3 technical replicates; two-tailed Student’s t-test; *p < 0.05; **p < 0.01; ***p < 0.001; bars represent the mean ± SEM). **(B)** LFD results of the complete Cas12a system and the absence of buffer, Cas12a, crRNA, reporter, or target DNA (T is the test band and C is the control band). 1 is the complete system, 2, is the system without buffer, and 3–6 are systems without LbCas12a protein, crRNA, reporter, and target DNA, respectively. **(C)** Cas12a cutting target DNA was detected by agarose gel electrophoresis. 1 is the complete system, 2–5 are systems without Cas12a, crRNA, buffer, and reporter, respectively, and 6 is the target DNA alone.

The LbCas12a protein is composed of 1,228 amino acids with a molecular weight of 143,035 g/mol. The results of SDS-PAGE analysis of purified LbCas12a protein were consistent with the expected size of the target protein (**Figure 2**). To verify the protein activity of LbCas12a, the target DNA (*ctxA*: 691bp, O1: 1033bp, O139: 965bp) was amplified by PCR and then added into the CRISPR reaction system to detect its fluorescent signal. The CRISPR reaction could detect fluorescent signals in the absence of buffer, but the fluorescence value was much lower than when the buffer is included (**Figure 3A**). This indicates that buffer significantly enhanced the accessory cleavage ability of the protein. The LbCas12a protein, crRNA, reporter, and target DNA were obligate for successful detection by the fluorescence method or the LFD method (**Figures 3A, B**). The CRISPR reaction products were detected by electrophoresis. The LbCas12a protein cleaved the target DNA successfully under the guidance of crRNA. Taken together, these findings indicated that *ctxA*-crRNA, O1-crRNA, and O139-crRNA were suitable for detection of the target DNA (**Figure 3C**).

### Specificity of CARID for Detection of *V. cholerae*

A total of 154 strains of *V. cholerae* were used to evaluate the specificity of CARID, and 112 toxigenic *V. cholerae* and 42 nontoxigenic strains were successfully identified. Forty-four serogroup O1 strains and 110 serogroup O139 strains were also successfully identified. The identification rate was 100%, which was consistent with whole-genome sequencing data. The RAA primers and crRNA of *ctxA*, O1, and O139 were used to detect other diarrheagenic bacteria, namely, *E. coli*, *Salmonella enteritidis*, *Shigella*, *Y. enterocolitica* and other Vibrios to confirm the specificity of the method. There were no positive results for these bacteria, and the coincidence rate was 100% (**Table 1**). The results of the fluorescence assay were consistent with those of the LFD.

**Table 1:** Specificity of CARID for detection of *V. cholerae* and other intestinal diarrheagenic bacteria.

Pathogens	Number	Positive rate		
		*ctxA*	O1	O139
Toxigenic serogroup O1 *V. cholerae*	22	22/22 (100%)	22/22 (100%)	0
Toxigenic serogroup O139 *V. cholerae*	90	90/90 (100%)	0	90/90 (100%)
Nontoxigenic serogroup O1 *V. cholerae*	22	0	22/22 (100%)	0
Nontoxigenic serogroup O139 *V. cholerae*	20	0	0	20/20 (100%)
*V. parahaemolyticus*	20	0	0	0
*V. alginolyticus*	14	0	0	0
*V. mimicus*	13	0	0	0
*V. furnis*	14	0	0	0
*V. fluvialis*	10	0	0	0
*E. coli*	14	0	0	0
*Salmonella enteritidis*	15	0	0	0
*Shigella*	14	0	0	0
*Y. enterocolitica*	15	0	0	0

### Sensitivity of CARID for Detection of *V. cholerae*

**Figure 4:**
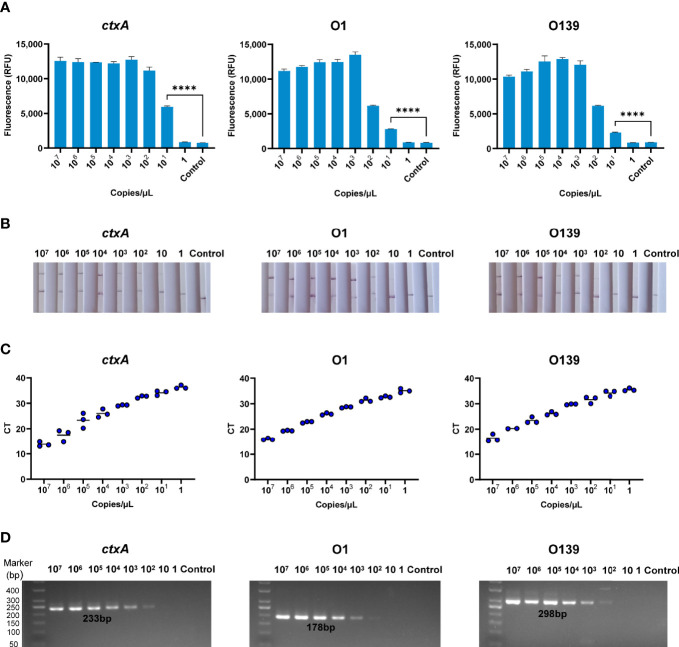
Sensitivity of CARID for detection of *V. cholerae*. **(A)** The 10^7^–1 copies/μl *V. cholerae* templates were detected by fluorescence method of CARID (n = 3 technical replicates; two-tailed Student’s t-test; ****p <0.0001; bars represent the mean ± SEM). **(B)** The 10^7^–1 copies/μl *V. cholerae* templates were detected by LFD. **(C)** QPCR detected *ctxA*, O1, and O139 genes from 10^7^–1 copies/μl *V. cholerae* templates. **(D)** PCR detected *ctxA*, O1, and O139 genes from 10^7^–1 copies/μl *V. cholerae* templates.

A series of samples containing *V. cholerae* genomic DNA that had been diluted from 10^7^ to 1 copies/µl were analyzed by CARID, PCR, and qPCR. Furthermore, the primers of RAA were used in the PCR experiment. The results showed that CARID could detect 20 copies/reaction (**Figure 4A**). However, only weakly positive or negative results were observed when samples containing 20 copies/reaction of genome DNA were tested by LFD (**Figure 4B**). When the genome concentration was 20 copies/reaction, the CT values of *ctxA*, O1, and O139 O-PS specific genes were 34.15 ± 0.92, 32.63 ± 0.40, and 34.19 ± 1.08 (mean ± SD), respectively, for qPCR (**Figure 4C**). Moreover, only weak bands were amplified by PCR at a genomic concentration of 200 copies/reaction (**Figure 4D**).

### Verification of CARID With Simulated Samples

**Figure 5:**
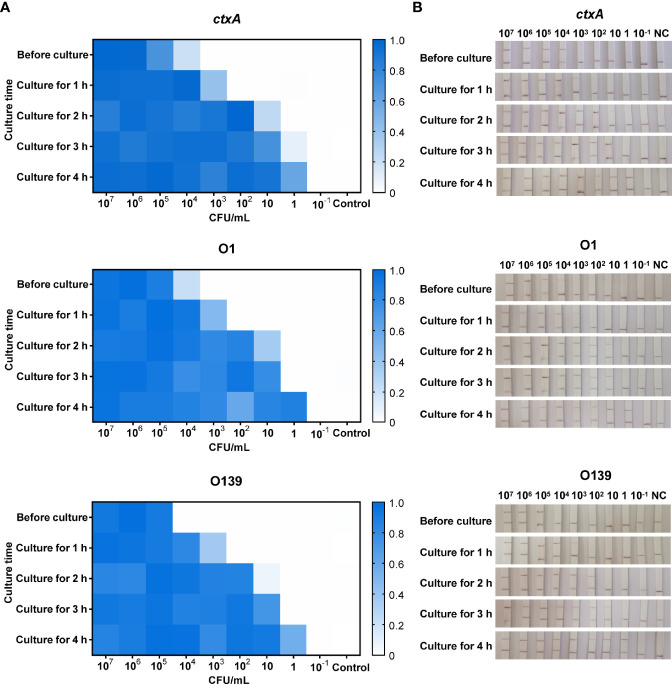
CARID detection of simulated river water samples. **(A)** Simulated water samples were tested by fluorescent CARID before culture and after culture for 1–4 h. The fluorescent signal of each sample was normalized against the negative control of the same batch. The heatmap represents normalized mean fluorescence values (n = 3 technical replicates). **(B)** Simulated water samples were tested by the LFD method of CARID before culture and after culture for 1–4 h.

A series of simulated river water samples containing 10^7^–10^−1^ CFU/ml toxigenic *V. cholerae* was used to evaluate the limit of detection (LOD) of CARID. For the toxigenic serogroup O1, positive results for *ctxA* and O1 O-PS specific genes were obtained in samples containing 10^4^ CFU/ml of *V. cholerae*. Samples containing 10^3^ CFU/ml could be detected after 1 h of culture, while 10 CFU/ml could be detected after 2 h of culture, and 1 CFU/ml could be detected after 4 h. In contrast to *ctxA* and O1, O139 O-PS specific genes could be detected in river water containing 10^5^ CFU/ml before culture and 10^2^ CFU/ml after 2 h of culture (**Figure 5**). Overall, river water samples containing 1 CFU/ml of *V. cholerae* could be successfully detected by CARID after culture for 4 h with alkaline peptone water.

**Figure 6:**
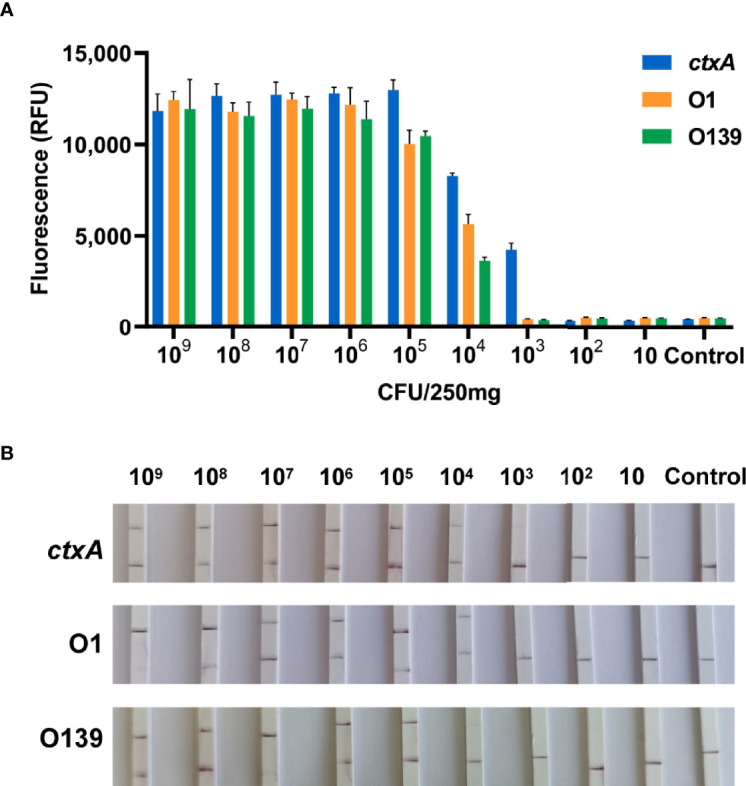
CARID detection of simulated stool samples. **(A)** Templates extracted from stool containing 10^9^–10 CFU/250 mg *V. cholerae* were analyzed by fluorescent CARID (n = 3 technical replicates; bars represent the mean ± SEM). **(B)** Templates extracted from stool containing 10^9^–10 CFU/250 mg *V. cholerae* were analyzed by the LFD method.

Application of CARID to stool samples containing 10^9^–10 CFU/250 mg bacteria revealed the *ctxA* gene could be detected at 10^3^ CFU/250 mg bacteria (equivalent to 4 × 10^3^ CFU/g). In addition, the O1 and O139 genes could be detected from simulated stool samples at 4 × 10^4^ CFU/g bacteria (**Figure 6**).

### Identification and Serotyping of *V. cholerae* With Multiple-CARID

**Figure 7:**
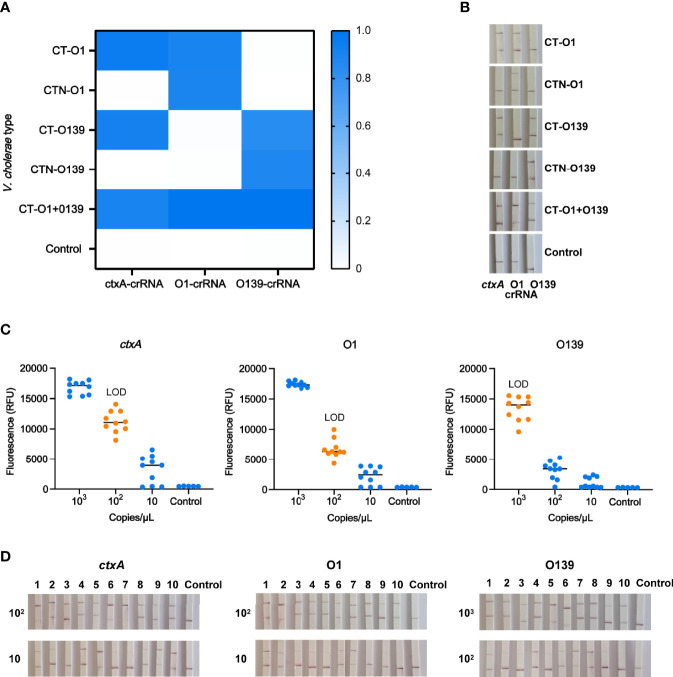
Identification and typing of *V. cholerae* with multiple-CARID. **(A)** Fluorescent CARID for detection of *ctxA* and identification of serogroups O1 and O139. The heatmap represents normalized mean fluorescence values (n = 3 technical replicates). CT-O1 refers to *V. cholerae* serogroup O1 containing cholera toxin; CTN-O1 refers to *V. cholerae* serogroup O1 without cholera toxin. CT-O139 refers to O139 containing cholera toxin; CTN-O139 refers to O139 without cholera toxin. CT-O1 + O139 refers to mixed *V. cholerae* serogroups O1 and O139 CT. **(B)** LFD method of CARID for detection of *ctxA* and identification of serogroups O1 and O139. **(C)** LOD of the fluorescent method of multiple-CARID. Ten repetitions were performed. **(D)** LOD of the LFD method of multiple-CARID.

Owing to efficiency and economic considerations, we set up multiple-CARID for simultaneous detection of *ctxA* and O-PS specific genes. Using the protocol described in the *Materials and Methods* section, a nucleic acid mixture of toxigenic *V. cholerae* serogroups O1 and O139 was analyzed, and positive results were obtained using all three CRISPR reactions (**Figures 7A, B**). To evaluate the LOD of multiple-CARID, 10^3^–10 copies/µl of *V. cholerae* genomic DNA was examined, and 10 repeats were performed for each dose. The lowest dose point with a 100% detection rate was taken as the LOD. For the fluorescence assay, *ctxA* and O1 O-PS specific genes showed higher sensitivity than O139, with 200 copies/reaction of *V. cholerae* DNA consistently detected in 10 out of 10 tests, and 20 copies/reaction detected in 7 out of 10 tests. However, an O139 O-PS specific gene was detected in 9 out of 10 tests of samples containing 200 copies/reaction of *V. cholerae* DNA (**Figure 7C**). When LFD analysis was conducted, *ctxA* and O1 O-PS specific genes were detected from samples containing 200 copies/reaction of *V. cholerae* DNA with clear bands in all 10 replicates. In comparison, O139 O-PS specific genes were detected in samples containing 2,000 copies/reaction of *V. cholerae* DNA with 100% detection rates **Figure 7D**.

## Discussion

Cholera often occurs in areas with poor sanitation, limited resources, and low testing capabilities, and *V. cholerae* serogroups O1 and O139 are the predominant agents of cholera epidemics (Deen et al., 2020). Accordingly, rapid field detection and pathogen identification are critical to controlling cholera. In the present study, we developed a visual detection method for toxigenic *V. cholerae* under isothermal conditions based on the principle of CRISPR-Cas12a. The developed method successfully detected toxigenic *V. cholerae* and identified serogroups O1 and O139 in less than 1 h, which is ideal for rapid responses in cholera control. The buffer can significantly improve the fluorescence value, which supported that the addition of a buffer with Mg^2+^ significantly improved the reaction efficiency of Cas12a (Wu et al., 2020). To confirm that the system works effectively, we set the detection time of the Cas12a reaction to 30 min; however, 10 min was found to be sufficient to produce robust results. Nevertheless, our findings indicate that the efficiency of the test could be improved in the future.

The specificity of CARID for detection of *V. cholerae* and other intestinal diarrheagenic bacteria was comparable to those of other methods. The detection limits were 20 copies/reaction and 200 copies/reaction, respectively, for fluorescent signal and LFD detection, which are comparable to the detection limits of PCR and qPCR. In areas of cholera outbreaks, polluted water is the main infectious medium (Rafique et al., 2016). In the present study, CARID could detect the target pathogens from simulated river water samples with the concentration of 10^4^–10^5^ CFU/ml *V. cholerae*, while the infection dose of *Vibrio cholerae* in humans is 10^8^–10^11^ cells (Nelson et al., 2009). Our results showed that, after 4 h of enrichment, *V. cholerae* at an initial concentration of 1 CFU/ml could reach the detection limit of CARID. Therefore, for water samples, we recommend an enrichment step, which can improve the detection rate. Patients with symptoms of cholera usually shed 10^7^–10^9^ cells of *Vibrio cholerae* per gram of stool (Nelson et al., 2009), and the detection limit of CARID is 4 × 10^3^ CFU/g of stool. Therefore, these findings indicate that CARID is sensitive enough for application in cholera control and for analysis of surveillance samples. The LOD of simultaneous detection of *ctxA*, O1, and O139 serogroup-specific genes in a single amplification was 200–2,000 copies/reaction, which was comparable to single amplifications. Hence, the multiplex protocol is both time-saving and economically efficient.

A major concern of the CRISPR-Cas-mediated detection protocol is that cross-contamination transfer of amplicons can lead to aerosol contamination; therefore, we recommend that the configuration system, addition of templates, and transfer of amplification products be conducted in different experimental areas. For example, in SARS-COV-2 detection with CRISPR-Cas12a, Chen et al. (2020) effectively avoided aerosol contamination of the amplicon by covering the isothermal amplification reaction solution with mineral oil and pre-adding CRISPR reagents to the tube lid, which avoided opening of the tube cap during the entire process Chen et al. (2020). In addition, lab-on-a-chip and microfluidics can also be used to develop portable, sensitive, and cost-effective biosensing systems for Cas12a-mediated detection (Khizar et al., 2020; Ramachandran et al., 2020). In such systems, the RAA and CRISPR reagents can be integrated onto a chip in advance, which enables the application of CRISPR-Cas-mediated steps in various settings, namely, clinics, mobile testing stations, and rural areas.

## Data Availability Statement

The original contributions presented in the study are included in the article/supplementary material. Further inquiries can be directed to the corresponding author.

## Author Contributions

BP conceived and designed the study. PL and JC contributed to the experiment. ZhenL contributed to the analysis of bioinformatics. PL and BP contributed to writing the manuscript. JZ, ZheL, and BK contributed to the design of the experiment. All authors listed have made a substantial, direct, and intellectual contribution to the work and approved it for publication.

## Funding

This work was supported by the National Science and Technology Major Project (2018ZX10714-002).

## Conflict of Interest

The authors declare that the research was conducted in the absence of any commercial or financial relationships that could be construed as a potential conflict of interest.

## Publisher’s Note

All claims expressed in this article are solely those of the authors and do not necessarily represent those of their affiliated organizations, or those of the publisher, the editors and the reviewers. Any product that may be evaluated in this article, or claim that may be made by its manufacturer, is not guaranteed or endorsed by the publisher.
