# Supplementary material for: Visual Identification and Serotyping of Toxigenic Vibrio cholerae Serogroups O1 and O139 With CARID
Source: Front Cell Infect Microbiol. 2022 Mar 31;12:863435. doi: 10.3389/fcimb.2022.863435 (PMC9008587; doi:10.3389/fcimb.2022.863435)
Supplement: Supplementary file 1 [file Table_1.docx]

Supplementary Material

**The optimal reaction concentration of LbCas12a protein and crRNA**

In order to determine the optimal reaction concentration of LbCas12a protein and crRNA, the fluorescence values of the protein or crRNA at the concentration of 0 to 500nM were collected. It was found that when the concentration of crRNA was kept at 100nM, the protein concentration at 100nM to 500nM showed a strong accessory cleavage ability. However, when the concentration of LbCa12a protein was kept at 100nM, the change of crRNA concentration had no significant effect on the accessory cleavage ability of LbCa12a protein. (Figure S1) In order to save costs and ensure the high activity of Cas12a protein, the final concentration of LbCas12a protein and crRNA was determined to be 100nM.

## Supplementary Figures


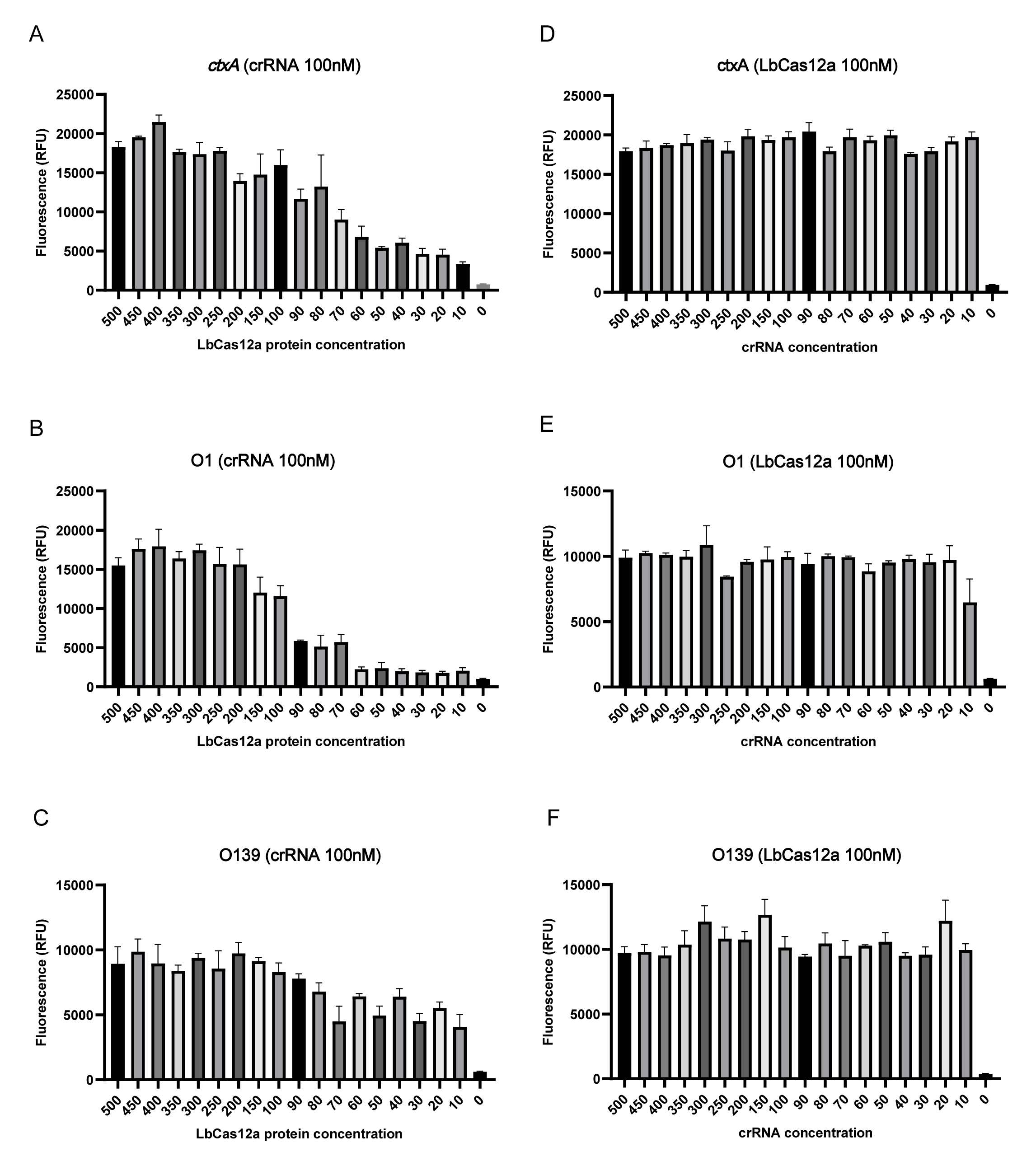


**Supplementary Figure 1.** The optimum concentration of LbCas12a protein and crRNA. (A,B,C) Fluorescence value of LbCas12a protein at 0-500nM when crRNA concentration is 100nM. (D,E,F) Fluorescence value of crRNA at 0-500nM when LbCas12a protein concentration is 100nM.
